# Virulence factors are preserved within carbapenem-resistant *Acinetobacter baumannii* clades

**DOI:** 10.1080/21505594.2025.2542489

**Published:** 2025-08-24

**Authors:** Mor N. Lurie-Weinberger, Adi Cohen, Hadas Kon, Ophir Kastel, Alona Keren-Paz, David Schwartz, Jonathan Lellouche, Amir Nutman, Sammy Frenk, Vered Schechner, Elizabeth Temkin, Lena E. Friberg, George L. Daikos, Anna Skiada, Emanuele Durante-Mangoni, Dafna Yahav, Leonard Leibovici, Yaakov Dickstein, Yael Dishon Benattar, Mical Paul, Yehuda Carmeli, On Behalf of the AIDA Study Group

**Affiliations:** a National Institute for Antibiotic Resistance and Infection Control, Ministry of Health, Tel Aviv, Israel; b Clinical Laboratories, Laniado Hospital, HaSharon, Netanya, Center District, Israel; c Faculty of Medicine, Tel Aviv University, Tel Aviv, Israel; d Department of Pharmacy, Uppsala University, Uppsala, Sweden; e First Department of Medicine, Laikon General Hospital, Athens, Greece; f School of Medicine, National and Kapodistrian University of Athens, Athens, Greece; g Department of Precision Medicine, University of Campania Luigi Vanvitelli, Napoli, Italy; h Department of Internal Medicine, AORN dei Colli-Monaldi Hospital, Napoli, Italy; i Infectious Diseases Unit, Sheba Medical Center, Ramat Gan, Israel; j Department of Medicine E, Rabin Medical Center, Beilinson Hospital, Petah Tikva, Israel; k Institute of Infectious Diseases, Rambam Health Care Campus, Haifa, Israel; l The Cheryl Spencer Department of Nursing, University of Haifa, Haifa, Israel; m The Ruth and Bruce Rappaport Faculty of Medicine, Technion – Israel Institute of Technology, Haifa, Israel

**Keywords:** *Acinetobacter baumannii*, antibiotic resistance, carbapenem resistance, virulence factors, evolution

## Abstract

Carbapenem-resistant *Acinetobacter baumannii* (CRAB) is an important threat to hospitalized patients. The virulence of *A. baumannii* varies between strains and geographic locations, which is believed to be related to its genetic plasticity. A comprehensive evaluation of virulence factor (VF) content of CRAB across genetically related clones is lacking. Therefore, we aimed to determine the evolution of VFs among 246 CRAB isolates belonging to major STs, ST2 and ST3. We used WGS to assess 136 VFs and found that 110 VFs were present in most isolates (49 VFs were universally present, 61 were ubiquitous) and 25 occurred sporadically. The distribution of the 25 sporadic VF genes was homogenous in ST3 but heterogeneous in ST2. Within ST2, we found high intra-clade homogeneity and high inter-clade heterogeneity of VFs. The homogeneity of VF content in ST2 clades and its uniformity in ST3 suggest a distant evolutionary segregation of VFs at the root of ST/clade divergence. VF content reflects remote events resulting in minimal variation between isolates belonging to the same ST/clade. Thus, the alleged differences in virulence of CRAB strains between geographical locations reflect differences in clonal distribution.

## Introduction

*Acinetobacter baumannii* is a nosocomial pathogen that is notorious for its ability to affect debilitated patients and cause severe and often fatal infections [1]. The species *A. baumannii* was designated in 1986 [2] and is recognized since the 1990s as a nosocomial pathogen. Over the last two decades, carbapenem-resistant *A. baumannii* (CRAB) has emerged as an important nosocomial pathogen of increasing incidence in various geographical areas. Because there are few antibiotics effective against CRAB, it is in the highest, “critical” category in the WHO Priority Pathogens List [3].

*A*. *baumannii* strains have a large pan-genome (over 8800 genes) and exhibit high genome plasticity [4]. The core genome is relatively small (about 2300 genes, mostly related to metabolic and general cellular processes), accompanied by a very large accessory genome, enriched in transport and transcription regulation functions [4–6]. CRAB emergence has been attributed to an increase in resistance and virulence of the pathogen, largely driven by lateral gene transfer via acquired plasmids and transposons [7]. An additional important determinant contributing to the success of *A. baumannii* is its ability to form biofilm that contributes to its long-term survival in the hospital environment, adherence to invasive devices, and protection from antibiotics and host immune responses [8].

The genome plasticity of *A. baumannii* has resulted in an exceptional ability to evolve and laterally acquire resistance to a variety of antibiotics, including β-lactams, tetracyclines, aminoglycosides, sulphonamides, and carbapenems [4,8], limiting the effectiveness of therapy.

Based on analysis of community-acquired and pre-antibiotic era isolates (isolated before 1940, when antibiotics were starting to be used on a large scale by humans) and comparison to more recent nosocomial isolates, it is believed that the initial *A. baumannii* population was genetically diverse. The introduction of antibiotics led to an evolutionary bottleneck, which resulted in reduced overall genetic diversity and persistence of only a few lineages [7,9]. CRAB is classified by two sequence-based MLST schemes: Pasteur and Oxford [10]. These methods allow for the division of *A. baumannii* isolates into evolutionarily related sequence types (STs). Over 2243 STs, grouped into several major clonal complexes (CCs), have been described (pubmlst). *A. baumannii* STs vary between geographical areas, with a single region or institution often containing several different CRAB STs simultaneously [7,11].

In a recent study, we found that ST2 and ST3 were the major CRAB clones in six hospitals in three Mediterranean countries (Israel, Italy, and Greece) [11]. These 149 ST2 isolates and 83 ST3 isolates were described in detail (MLST typing using the Pasteur scheme, KL and OCL), and a phylogenetic tree based on all isolates’ pangenome was constructed with all ST types available at that time (BioProject PRJNA640273).

ST2 is an important high-risk international clone, geographically widely dispersed, that has been reported in over 30 countries [7,11,12] and is the most prevalent ST type in numerous studies [13–15]. ST3 is far less common, reported from only eight countries, including Lebanon, Italy, and Israel [7,11,15,16]. Both clones are associated with high levels of antimicrobial resistance and virulence and belong to IC-I. Although IC-I has been reported to be monophyletic [17,18], isolates belonging to ST2 were found to differ significantly from each other in gene content and phenotypes. As MLST gives a very narrow view of genome diversity, we and others [5,11] suggested a pangenome phylogeny-based division of ST2 into eight different clades (2A–2H). In contrast, ST3 was found to be a single, monophyletic clone. We also described the MLST, antibiotic resistance genes, and plasmid content of these isolates [11].

In spite of many virulence factors (VF) being present in the core genome of *A. baumannii*, virulence varies between isolates. This may be the result of a multifactorial and combinatorial virulence strategy, similar to that of *P. aeruginosa*, which is based on a wide array of accessory genes and differential gene regulation [7]. Thus, the genetic diversity and combinatorial virulence of *A. baumannii* create a strong adaptive potential, contributing to this pathogen’s ability to cause serious infections [1,7].

The emergence of multi-drug resistant (MDR) *A. baumannii* as an important nosocomial pathogen causing severe infections may suggest co-evolution of resistance and virulence. Here, we aimed to examine the evolution of virulence factors and their association with the evolution of antibiotic resistance within CRAB clones and clades. We conducted an in-depth genomic analysis of our large collection of isolates causing severe infections and belonging to ST2 and ST3 and compared them to publicly available CRAB genomes.

## Materials and methods

### Study isolates

A total of 246 *A. baumannii* isolates were collected during the AIDA study, a multicenter randomized controlled trial of patients with severe infections, as previously described [19]. Of these isolates, 99 caused bacteraemia and 147 caused ventilator-associated or hospital-acquired pneumonia. Most AIDA isolates (*n* = 232) were previously sequenced and typed [11]; 14 other AIDA isolates were newly sequenced for this study.

For those 14 isolates, whole genomic DNA was extracted from a loop of overnight colonies resuspended in 100 µl phosphate-buffered saline (PBS). A total of 90 µl of Bacterial Lysis Buffer and 10 µl of Proteinase K (Qiagen GmbH, Germany) were added, incubated for 10 min at 65°C then 10 min at 95°C, and high-molecular-weight DNA was isolated using the MagLEAD 12gC (PSS co., Matsudo, Chiba, Japan) and MagDEA DX Sv Kit, according to manufacturer’s instructions. Resulting nucleic acids were sequenced at the Sequencing Core at the University of Illinois, Chicago. Genomic libraries were prepared using the Nextera XT (Illumina Inc., CA, USA) kit and sequenced using the high output 2X 150bp kit (Illumina Inc.). Assembly, antibiotic resistance genes (ARGs) detection and MLST typing were performed as previously described [11].

## Genomic data preparation and analysis

A list of the major virulence factors was extracted from the VFDB database (May 2022). A database of all genomes was constructed, and the presence/absence of each gene was tested using DIAMOND [20]. Genes were then plotted using R package GGTREE against a phylogenetic tree to assess patterns [21]. Subsequently, genes were further clustered using k-means clustering, without taking clades into consideration, using the pheatmap R package. The sequence of all *bap* genes was extracted, and a BlastN algorithm was used to compare the different sequences. The *bap* was then visualized against the VFDB reference, and the length of the protein was assessed and compared among all isolates to determine the missing portion. The cgMLST for each isolate was checked using PubMLST cgMLST scheme V1 (https://pubmlst.org/), and a profile data was used to create a tree with https://www.phyloviz.net/.

## Statistical analysis

We tested the differences in mean VF number between ST2 and ST3 using the t-test and between ST2 clades using the F-test, followed by post-hoc Tukey comparisons. We compared the variance of VFs in ST2 versus ST3 using the F-test for variance. We used the chi-square for independence test to assess whether a VF’s being sporadic or ubiquitous was associated with its designation as core or accessory. We tested for differences between the patterns of the clades (i.e. proportions of presence of specific VFs or ARGs) between clades. We used a paired t-test between each two clades with an adjusted *p*-value for multiple comparisons. Analyses were performed in RStudio version 2023.06.0 + 421. Cohen’s kappa test for each combination of VF and ARG, with adjusted *p*-values for multiple comparisons using the Benjamini-Hochberg False Discovery Rate

## Pan-genome analysis

Genomes of ST2 and ST3 from our sample and 100 publicly available ST2 genomes from https://gtdb.ecogenomic.org/ were analysed using Roary version 3.12.0. We did not analyse external ST3 genomes due to the small number of genomes publicly available. The resulting core gene alignment was used to build a phylogenetic tree with RAxML version 8.2.12 with the GTR gamma model. The resulting presence/absence of genes matrix and pie chart were plotted using a python script from the Roary package.

## Biofilm formation assay

Cultures grown overnight in Brain Heart Infusion (BHI) medium at 35 ± 2°C with shaking were diluted 1:1000 in fresh BHI medium and normalized to a bacterial concentration of OD600 0.1. Aliquots of 150 μL were inoculated in four replicates into a 96-well polystyrene microtiter plate (Nunc MicroWell 96-Well Microplates, Thermo Scientific, Waltham, MA, USA) and incubated at 35 ± 2°C without shaking for 24 hours. Suspensions were removed and wells were washed three times with sterile distilled water and then stained with 0.5% crystal violet solution (Merck, Rehovot, Israel) for 10 minutes. After removal of the dye and three washes with sterile distilled water, 95% ethanol (molecular grade, 95%, BioLab, Jerusalem, Israel) was added to the wells. The biofilm biomass was quantified by measuring OD540, and means were calculated. Isolates’ biofilm production level was classified as low, medium, or high by dividing the biofilm biomass into tertiles. We used a chi-squared test to determine whether the distribution of biofilm tertiles differed between ST2 and ST3.

## Results

A total of 246 *A. baumannii* isolates, 149 belonging to ST2 and 97 to ST3, were analysed (Supplementary Table S1). All but 14 isolates have been previously sequenced (Supplementary Table S1). These ST2 isolates had been previously divided into eight clades (2A–H) [11]. Here, we correlated these clades with cgMLST allelic profiles and found high concordance with the described clades (Supplementary Figure S1).

### Distribution of virulence factors

We explored the presence of the 136 major VFs in *A. baumannii* listed in the Virulence Factor Database (VFDB) [22].

The 136 virulence factors were divided into three groups (Supplementary Table S1): (1) 49 were universally present in all isolates, (2) 62 were ubiquitous (present in at least 200, i.e. >80% of the isolates), and (3) 25 were sporadic, missing in at least 20% of strains (including 4 that were universally absent (*tse2–4*)). We used the scheme of [6] to divide genes into core and accessory. Out of the 136 VF genes, 49% belonged to the core genome and 51% were part of the accessory gene pool. Among the 111 ubiquitous and universally present VF genes, 66 (59.5%) belonged to the core genome and 44 (39.5%) to the accessory genome. In contrast, all 25 (100%) sporadic VF genes were part of the diverse and adaptive accessory gene pool (*p* < 0.001) (Supplementary Table S2).

**Figure 1.**
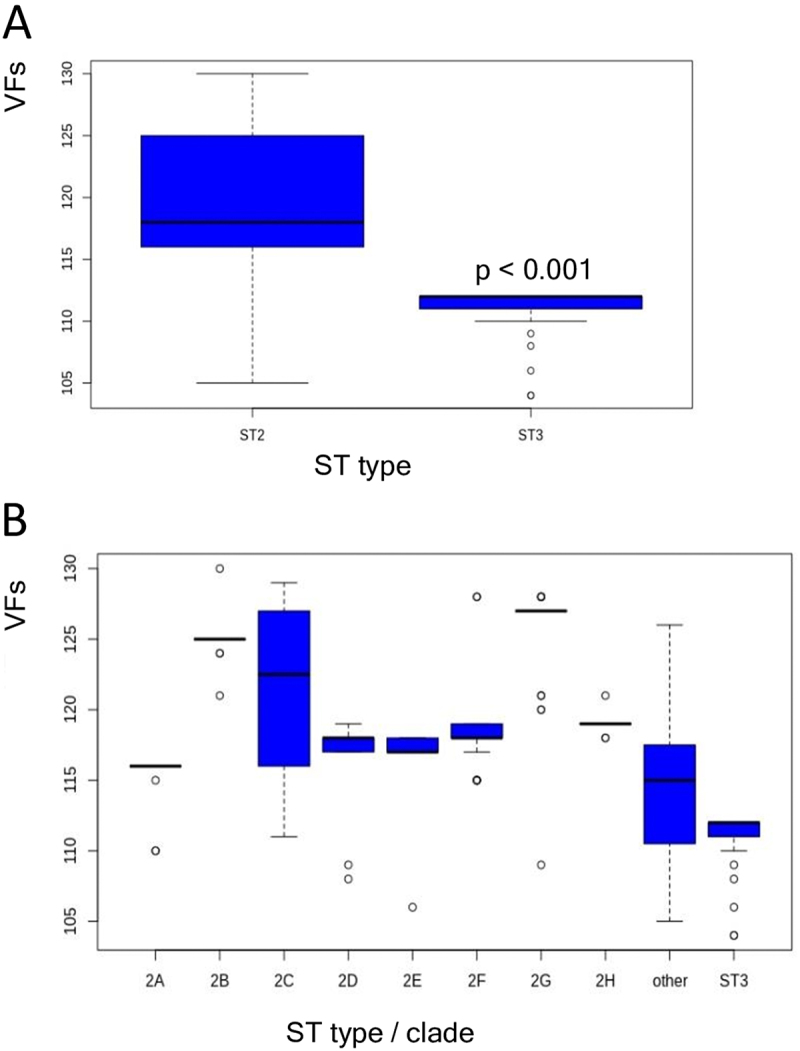
Virulence factors by sequence type (ST) and clades. A. Boxplot of number of VFs in ST2 and ST3 isolates. B. Boxplot of number of VFs in ST2 clades. Dots indicate outliers.

ST2 isolates had more virulence factors than ST3 isolates (a mean of 119.6 and 111.4, respectively, *p* < 0.001) (Figure 1A, Supplementary Table S1). ST2 VF composition was more diverse, while ST3 was homogenous (*p* < 0.001). Because ST2 is not monophyletic, we examined VF composition by clades. ST2 clades had distinctly different average virulence factor content. One group comprising clades 2A, 2D and 2E had an average of 115.4, 116.5 and 116.5 VF genes, respectively, whereas a second group comprising clades 2G and 2B had a higher average number of VFs, 125.5 and 124.9, respectively (Figure 1B). There was a significant difference in the average number of VFs between these two groups (*p* < 0.001). There was significantly higher uniformity of gene content within clades compared to that between clades (*p* < 0.001), indicating a shared evolutionary history of VFs in the same clade.

To examine the diversity in VFs among the 149 ST2 isolates, we focused further analysis on the 25 sporadically occurring genes. The association between the clades and the VF distribution patterns was statistically significant (*p* = 0.00493, determined by the F-test). Each clade displayed a similar pattern of VF content, with minor variations in one or two genes. The exception was clade 2C, which had a distinct composition. Within the 2C clade, two VF gene clusters were observed, corresponding to the phylogenetic branches of this clade. Specific pairwise comparisons revealed significant differences in VF patterns: 2G vs. 2A (*p* < 0.01), 2G vs. 2E (*p* < 0.05), 2A vs. 2B (*p* < 0.05), 2B vs. 2E (*p* < 0.05), 2G vs. 2F (*p* < 0.05).

All ST2 isolates contained three genes forming a shared backbone – *bap, bauA*, and *galE*. The gene *bauA* encodes for a TonB-dependent siderophore receptor, *galE* encodes for UDP-glucose 4-epimerase related to capsule construction, and *bap* is biofilm-related. A secondary group, containing RS004605 encoding for a YbaN family protein, as well as type VI secretion system genes *tssB* and *tssI* (*vgrG_1*), forms a secondary backbone in ST2, present in most, but not all, isolates.

**Figure 2.**
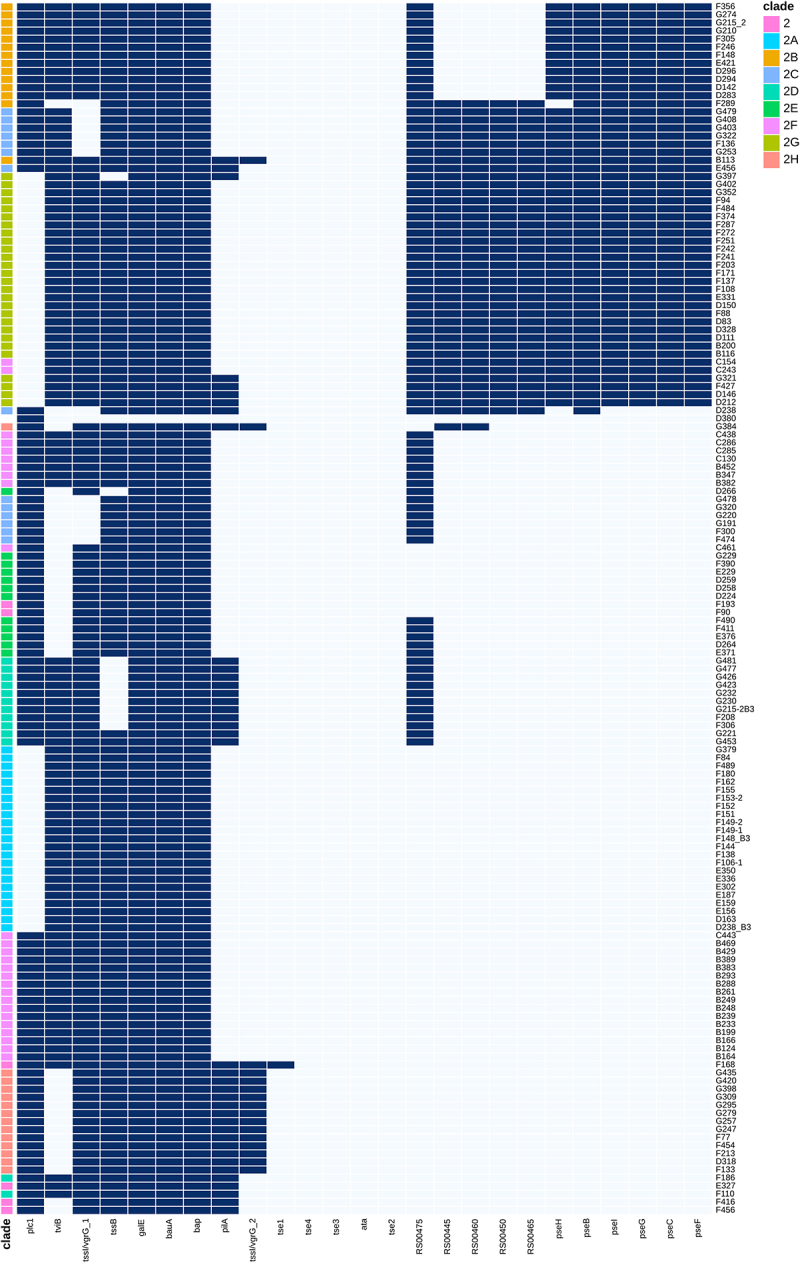
Clustered heatmap of sparse virulence factors among ST2 isolates. Blue boxes indicate that the gene is present; white boxes show the gene is absent. The colours on the left indicate clades. Isolates marked as “2” are ST2 with no designated clade.

Other VFs varied between the clades. For example, *plc1* was missing from both 2B and 2G clades but was commonly present in all other clades. The 2B and 2G clades differed in sporadic VF content: 19 of the 25 sporadic VF genes were absent in clade 2B, while in clade 2G, fewer VFs (6–7 genes) were absent in each isolate. Likewise, the genes *tssI/vgrG_2* and *pilA* were present in all clade 2H isolates but were rare among other clades (Figure 2).

To further test VF patterns, we generated a clustering analysis of the 25 sporadic genes, where isolates were ordered by the similarity of gene patterns regardless of their clade. Clustering analysis showed that the isolates could be divided into two groups by the presence of six genes related to non-2-ulosonic acid synthesis (*pseBCFGHI*) (Figure 2). All clades but 2C had either all six genes or none. These results correspond to the phylogenetic tree that showed that clade 2C was comprised of two distinct sub-clades. This group containing *pseBCFGHI* could be further divided into two sub-groups: sub-group 1, which contained the capsule-related genes encoding for RS00445, RS00450, RS00475, and RS00460 and included one 2B (B113), 8/14 2C, and 27/27 2G isolates and sub-group 2, which was missing the complete set and was comprised of all 2B isolates except for B113.

**Figure 3.**
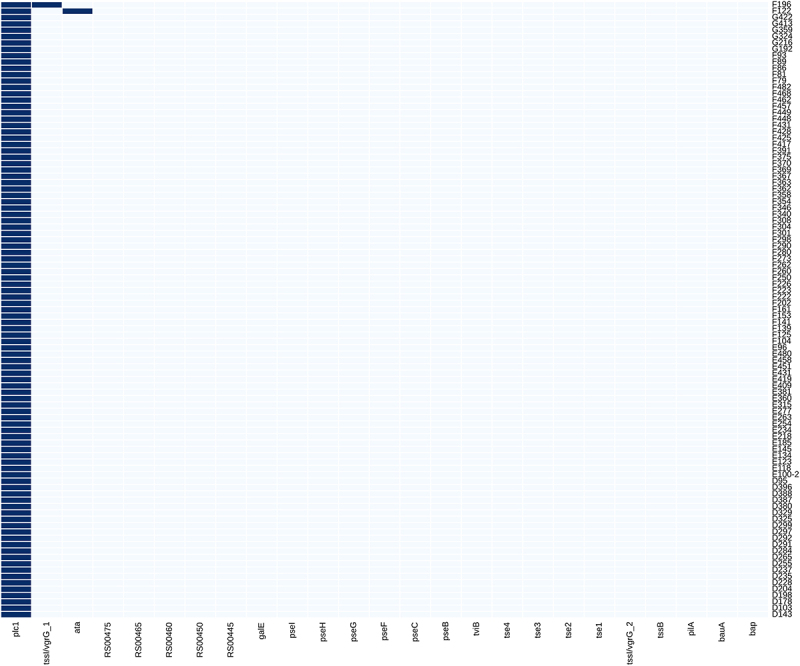
Clustered heatmap of sparse virulence factors among ST3 isolates. Blue boxes indicate that the gene is present; white boxes show the gene is absent.

In contrast to ST2, ST3 showed a homogenous VF pattern, with almost all isolates sharing the same composition of the 25 sporadic genes. Only 2 of the 97 did not fit this pattern, each varying by a single gene: isolate F122 had the additional gene *ata*, and isolate F196 carried the additional gene *tssI/vgrG_1* (Figure 3). This result supports the hypothesis that VFs in ST3 isolates have a common evolutionary background, clearly distinct from ST2. Cluster analysis confirmed this result shown in the heatmap (Figure 3). One striking example of these uniform patterns was the absence of the *bap* gene. The *bap* gene, encoding biofilm-associated protein BAP, was present in all ST2 strains, with the majority of isolates (irrespective of their clade) harbouring the same 6069 bp long *bap* variant that encodes a protein of 2022 aa. In contrast, none of the ST3 isolates harboured the *bap* gene (Figure 2 and 3). However, we identified a novel 4239 bp gene encoding a 1412 aa long protein present in all ST3 isolates. This gene contained Ig-like repeats and shared 74% identity with *bap*. BlastP comparison between the reference *bap* (ACICU_RS14670) revealed 47% aa identity, mostly in the C terminus (except for the first 73 amino acids of the reference), followed by an identity rate of 86% (207/240) for the next 240 amino acids. The NH_2_ portion was missing. This new gene shared no similarity in sequence with *blp1 and blp2* (which encode BAP-like proteins 1 and 2), which also feature Ig-like repeats and share a sequence motif at the NH_2_ terminus with *bap*. We designated the gene as *blp3*. Phenotypic analysis revealed that ST3 isolates formed on average 7% less biofilm when compared to ST2 isolates, a difference that is not statistically significant (Supplementary Table S3). In addition, when we classified isolates as having low, medium, or high biofilm biomass, ST2 and ST3 did not differ significantly (*p* = 0.61). Another gene that differed between ST2 and ST3 is *plc1*. All ST3 isolates carried the gene, as opposed to only 97 (65%) of ST2 isolates.

### VF pattern in publicly available ST2 isolates

We randomly selected 100 publicly available ST2 isolates from 15 countries representing different continents and analysed their VF content (Supplementary Table S4). Among these 100 isolates, 31 genes were universally present, 77 were ubiquitous, and 28 were sporadic. The average number of VFs was 113.9, with a wide range (87–130) between isolates. Here again, clustering of the 25 sporadic VFs revealed two large groups differing by the presence of genes related to non-2-ulosonic acid synthesis (Supplementary Figure S2). This group could be further divided into two sub-groups: one that contained capsule-related genes and one that did not (Supplementary Figure S2). We created a pan-genome-based phylogenetic tree of the 100 publicly available genomes, as well as a representative from each of the eight ST2 clades in our sample (Supplementary Figure S3). This figure shows that some of the patterns of sparse VFs that we detected in our sample also exist in other countries (Supplementary Figure S3).

### Pan-genome analyses

We performed a pan-genome analysis for each of the three groups: 149 ST2 genomes from our sample, 100 randomly selected publicly available ST2 genomes, and 97 ST3 genomes from our sample (Supplementary Figure 4). The pan-genome of ST3 indicated a very homogenous genomic content, while both ST2 pan-genomes were much bigger in size and showed greater genomic variety. ST3 isolates shared 3180/4698 (67.5%) while ST2 isolates from our sample had a wider variety of gene pools, with only 2865/6962 (41%) genes as part of the core. The publicly available ST2 genomes displayed the greatest diversity, with only 2183/10,988 (<20%) being core.

### Association between VF and ARG distributions

We have previously reported 57 sparse ARGs present in the study isolates [11]. Using the same statistical analysis as for VFs, we tested ARG distribution proportions and patterns (Supplementary Table S5). The association between the clades and the ARG distribution patterns was not statistically significant, both overall and between each clade pair (*p* > 0.05). We examined the association between the occurrence of ARGs and VFs and found that among the 1425 possible combinations, only 71 had a statistically significant substantial correlation, and 1354 (95.02%) did not (Supplementary Figure S5). Among the 71 correlated pairs, all but 5 were a combination of ARGs and VFs that are clone or clade specific (Supplementary Figure S5).

## Discussion

*A*. *baumannii* has emerged over the last two decades as an important human pathogen with exceptional survival mechanisms [7,23]. Though mostly discussed through the narrow scope of antibiotic resistance, *A. baumannii* has other attributes that allow for a “persist and resist” strategy that utilizes diverse virulence factors [23]. Several studies have tried to better understand *A. baumannii*‘s virulence inside and outside the clinical setting [17,24–27]. Differences in the virulence of *A. baumannii* between geographical areas and strains are often described. It is believed that similar to antibiotic resistance, these differences are related to the exceptional genome plasticity of *A. baumannii* and its enormous and diverse accessory genome.

Here, we focused our analysis on two of the largest and most important *A. baumannii* groups, ST2 and ST3 [9]. We previously described that ST2 and ST3 differ in their content of ARGs, capsular lipooligosaccharide outer core proteins, and plasmids [11]. Here, we extended our analysis to study the distribution of virulence factors among this large collection of clinical CRAB isolates belonging to ST2 and ST3, in order to understand the evolutionary history of VFs.

We found that the majority of the isolates had a rich VF arsenal. Out of 136 VFs examined, the study strains carried between 106 and 129 virulence factors each. Importantly, we found that the majority of the VFs (111/136, 81.6%) were ubiquitous. Only 60% of these ubiquitous VFs were members of the core genome of *A. baumannii* [6], while the other were part of the accessory genome, suggesting that they were acquired in the far past by a common distant evolutionary ancestor. Indeed, Antunes et al. noted that most *A. baumannii* VFs are conserved in the *Acinetobacter* complex and suggested that *A. baumannii* contained them before its emergence as a pathogen [7]. We found a subset of 25 genes more sparsely distributed among the study isolates (sporadic VFs), thus representing more recent evolution. All these genes were members of the accessory genome. An additional analysis of 100 randomly selected ST2 genomes from 15 countries, not selected based on infection severity, indicated that the sporadic VF pattern is similar across territories and among isolates from severe infections and those from an unselected sample. The two groups of isolates, one with and one without six non-2-ulosonic acid synthesis genes (*pseBCFGHI*), detected in our sample, were also present worldwide. A subgroup of these isolates that contained the capsule-related genes encoding for RS00445, RS00450, RS00475, and RS00460 genes was also globally present. Thus, there is evidence that even on a global scale, VF patterns are conserved within monophyletic clades.

We found that the 25 sporadic virulence factors markedly differ between ST2 and ST3 isolates, with one gene, *bap*, completely segregated between the two sequence types. We also revealed that the VF composition in ST3 is uniform, while in ST2, the sporadic VF distribution is heterogeneous across the ST but homogeneous within each clade. This finding indicates that the ancient ancestor of each clade was already equipped with these sporadic VFs.

We found that ARG and VF compositions are independent of each other. Only 5% of possible ARG and VF combinations were substantially correlated. Most of these pairs included ARGs (*tet(A)* vs. *tet(B), sul1* vs. *sul2* and *ampC* alleles) that were previously reported as corresponding to a specific ST or clade [11]. Here, we showed that there is a far greater diversity in ARG content within STs and clades than that of VFs. Indeed, 40 of 57 ARGs were present in fewer than 20% of isolates, indicating that, in contrast to VFs, ARG variability is independent of ancestral origin. This diversity points to a more recent evolution of ARGs, independent from the evolution of VFs and suggests that once a specific clone, equipped with a set of VFs, becomes successful, it is subject to rapid resistome changes through lateral gene transfer.

Our results support the hypothesis that each clade is a descendant of a single, successful ancestor with a set array of virulence genes and some antibiotic resistance determinants that disseminated over time. More recent events of lateral gene transfer, a hallmark of *A. baumannii*’s extreme plasticity and an important factor in its adaptability to diverse environments [7], are mostly limited to antibiotic resistance genes, and not to VFs, and are therefore only partially responsible for the success of *A. baumannii* as an important pathogen.

Our findings suggest that some of the sporadic VFs may be essential for the success of ST2 and ST3 CRAB as a nosocomial pathogen. For example, biofilm-associated protein (BAP) is an important determinant of biofilm formation and adhesion to host cells [8] and is necessary for mature biofilm formation on environmental surfaces [28]. While all ST2 isolates carried *bap*, it was absent from all ST3. However, we found that all ST3 isolates carried a novel BAP-like protein gene, *blp3*, which was absent in ST2. This suggests that these two gene homologs may be essential to the success of the clones as nosocomial pathogens. Similarly, we identified a backbone of sporadic VFs that are preserved among all ST2 isolates across clades, suggesting that they are important to this ST’s success.

Our study has several limitations. First, isolates were collected in three Mediterranean countries and therefore may not be representative of all CRAB population worldwide. Also, our isolates were collected from patients with severe infections; therefore, other less virulent CRAB populations may not have been captured.

It is commonly accepted that CRAB’s genomic plasticity is a key factor in its evolution. Our findings show that the evolution of *A. baumannii* is multidimensional. The VF content of each isolate reflects distant evolutionary events, thus resulting in minimal variation between isolates belonging to the same ST or clade. In contrast, ARG composition reflects more recent events and therefore, displays a higher between-isolate variation. In order for a pathogen to become successful in the clinical setting, it requires both VFs and an ability to survive the selective pressures of an antibiotic-rich environment. Thus, both the ancestral origin, determining its virulence, and more recent lateral gene transfer, determining its resistance, are essential. Consequently, the emergence of a new multidrug resistant clade is a complex and rare event. Surveillance to detect such events can prompt interventions to prevent new clades from establishing endemicity.

## Conclusions

In CRAB, the majority of VFs are preserved across STs and clades. Sparsely occurring VFs form homogenous patterns within clades, this suggests their distant ancestral origin and slow evolution, i.e. VF content is retained from the original successful clones from which they arose. Our results support the theory that the formation of new virulent CRAB clones is uncommon. This is in contrast to ARGs, whose diversity within clades indicates more recent, ongoing evolution.

## Ethics

The AIDA trial was approved by the Institutional Reviews Boards of the participating hospitals. Written informed consent was obtained from participants according to local requirements. Access to the 100 publicly available genomes from https://gtdb.ecogenomic.org/ that were analysed in this study was permitted via an open licence. The Tel Aviv Sourasky Medical Center Institutional Review Board determined that analysis of all bacterial genomes is exempt from review because it does not involve human subjects.

## Supplementary Material

Supplementary_Figure_4.tiff

Supplementary_Figure_1.tiff

Supplementary_Figure_2.tiff

Supplementary_Figure_3.tiff

Supplementary_Figure_5.tiff

## Data Availability

All sequence data were deposited at the NCBI repository under bioproject PRJNA640273. Specific biosample accession numbers are listed in Supplementary Table S1. The sequence for *blp3* is available under accession number PQ568385. Supplementary Tables are available under accession number Lurie-Weinberger, Mor (2025), “Virulence_Genomic_diversity_of_CRAB,” Mendeley Data, V1, doi: 10.17632/8st9mf4smd.1
