# Supplementary figures and images for: Virulence factors are preserved within carbapenem-resistant Acinetobacter baumannii clades
Source: Virulence. 2025 Aug 24;16(1):2542489. doi: 10.1080/21505594.2025.2542489 (PMC12377095; doi:10.1080/21505594.2025.2542489)

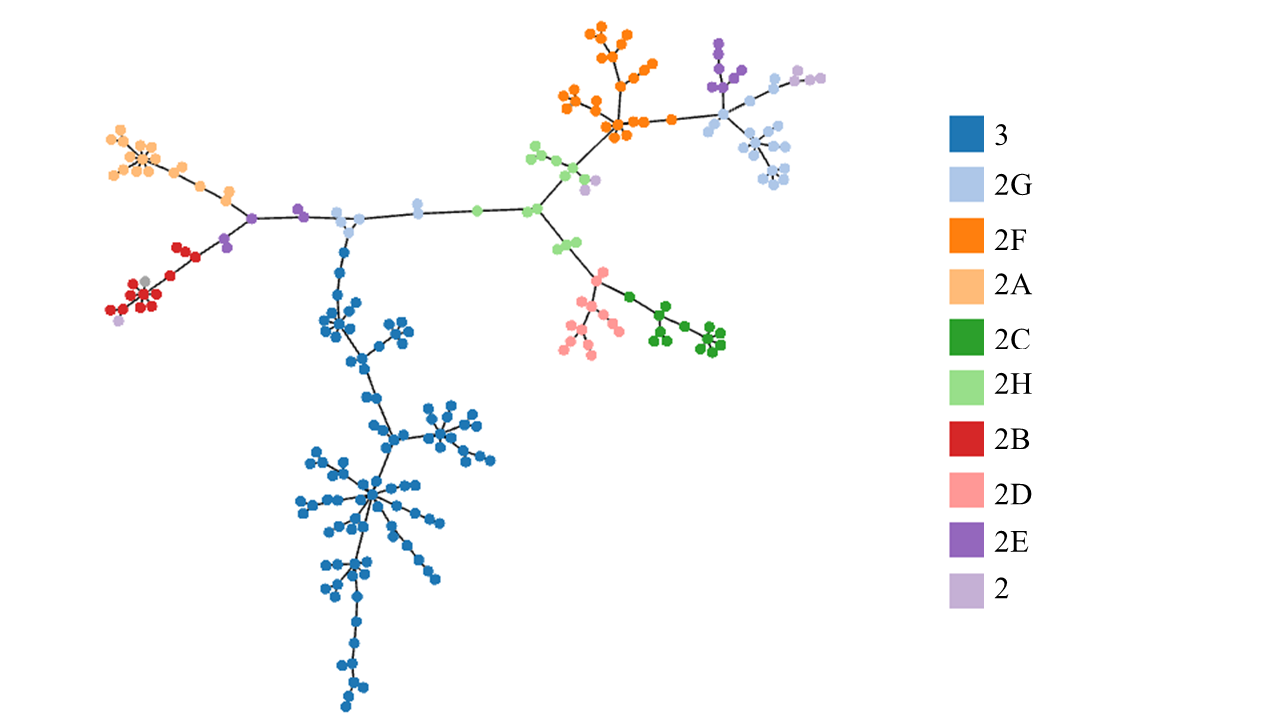

Supplement: Supplementary_Figure_1.tiff [file KVIR_A_2542489_SM9162.tiff]

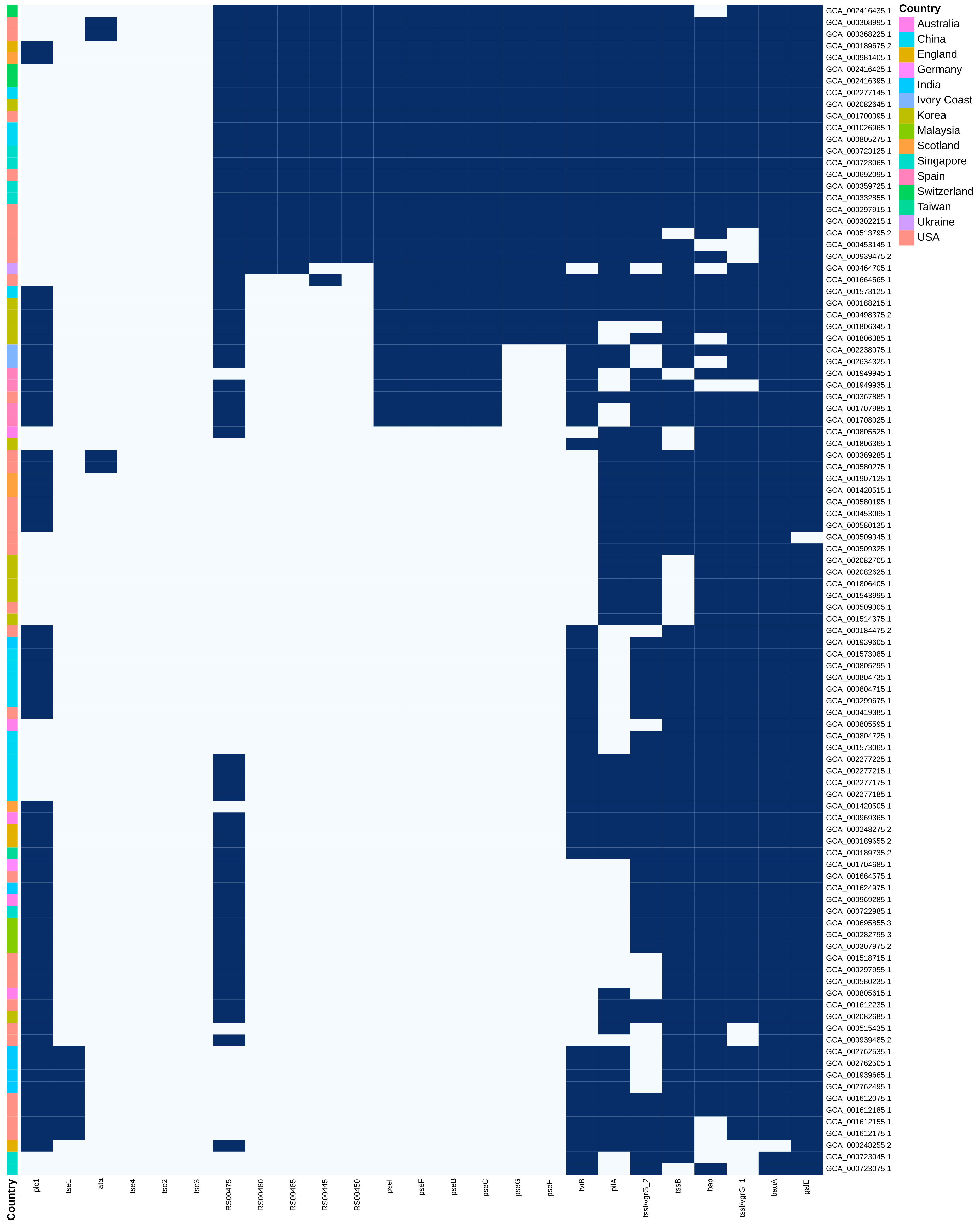

Supplement: Supplementary_Figure_2.tiff [file KVIR_A_2542489_SM9161.tiff]

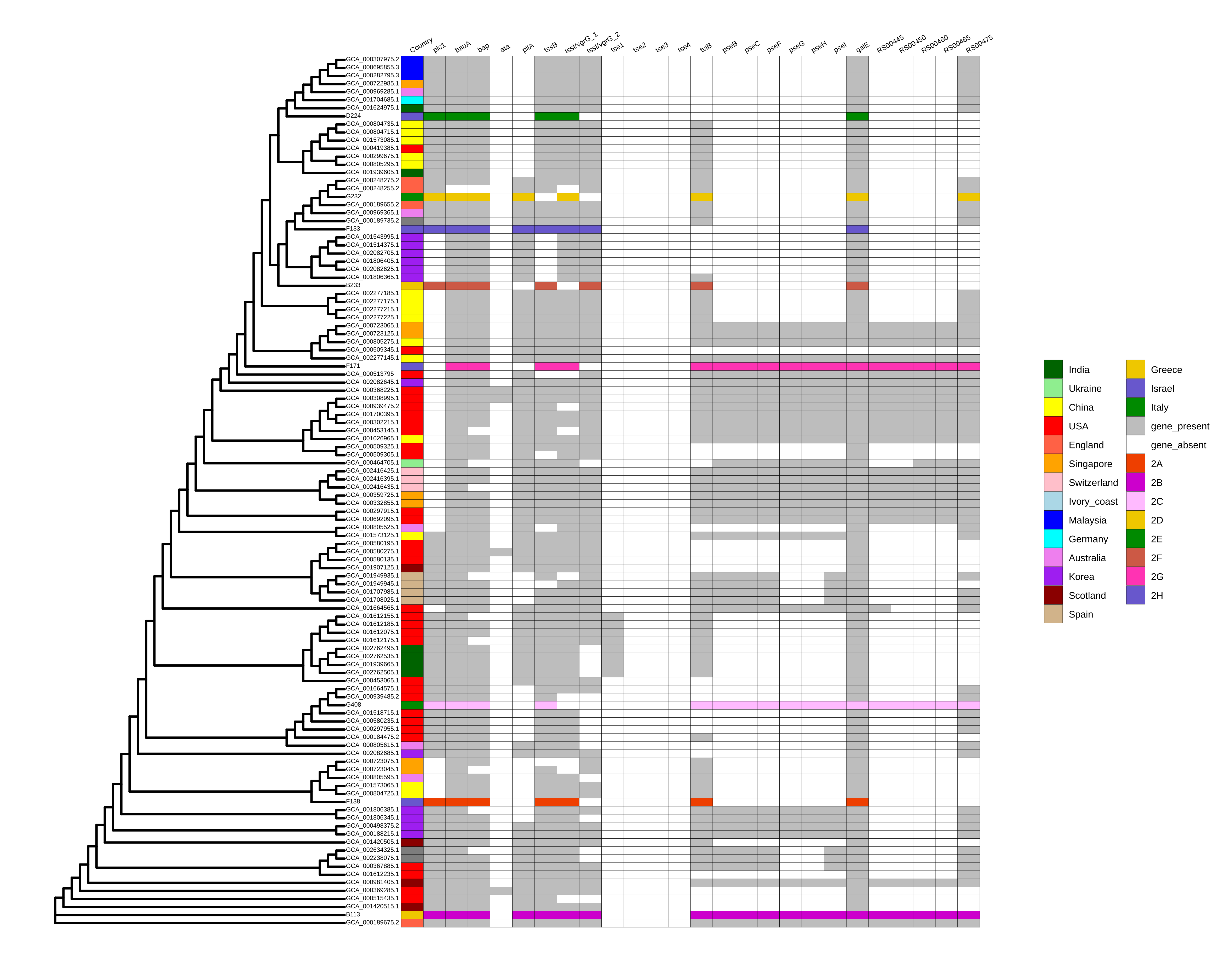

Supplement: Supplementary_Figure_3.tiff [file KVIR_A_2542489_SM9160.tiff]

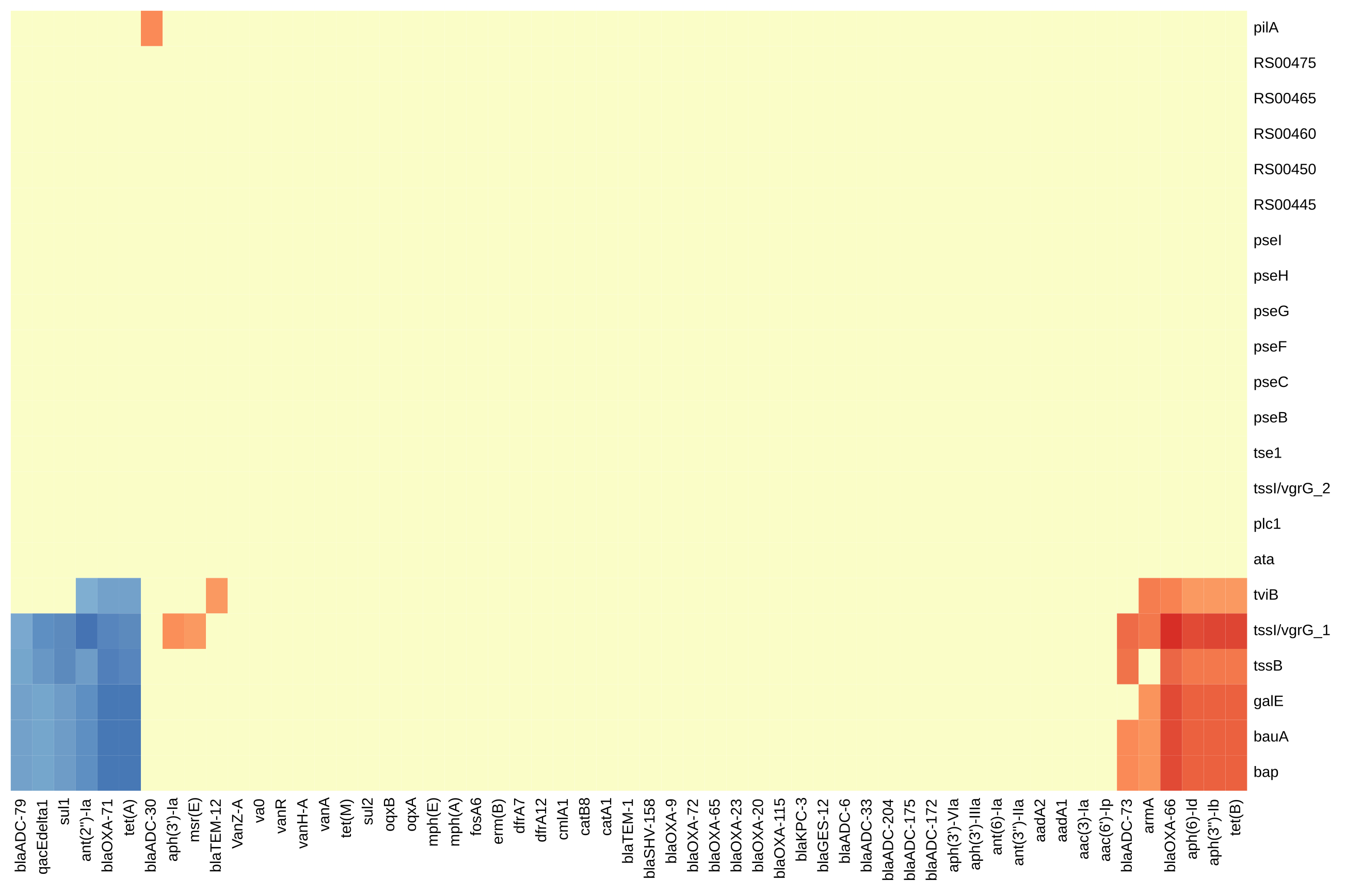

Supplement: Supplementary_Figure_5.tiff [file KVIR_A_2542489_SM9159.tiff]
